# Dissection of Myogenic Differentiation Signatures in Chickens by RNA-Seq Analysis

**DOI:** 10.3390/genes9010034

**Published:** 2018-01-11

**Authors:** Tingting Li, Genxi Zhang, Pengfei Wu, Lian Duan, Guohui Li, Qiuhong Liu, Jinyu Wang

**Affiliations:** 1 College of Animal Science and Technology, Yangzhou University, Yangzhou 225000, China; d150070@yzu.edu.cn (T.L.); Wu_P_Fei@163.com (P.W.); qiuhongliu@163.com (Q.L.); 2 Jiangsu Institute of Poultry Science, Chinese Academy of Agricultural Science, Yangzhou 225000, China; yzduanlian@163.com (L.D.); sahui2008@163.com (G.L.)

**Keywords:** RNA sequencing, lncRNA, mRNA, myoblasts, chicken

## Abstract

A series of elaborately regulated and orchestrated changes in gene expression profiles leads to muscle growth and development. In this study, RNA sequencing was used to profile embryonic chicken myoblasts and fused myotube transcriptomes, long non-coding RNAs (lncRNAs), and messenger RNAs (mRNAs) at four stages of myoblast differentiation. Of a total of 2484 lncRNA transcripts, 2288 were long intergenic non-coding RNAs (lincRNAs) and 198 were antisense lncRNAs. Additionally, 1530 lncRNAs were neighboring 2041 protein-coding genes (<10 kb upstream and downstream) and functionally enriched in several pathways related to skeletal muscle development that have been extensively studied, indicating that these genes may be in *cis*-regulatory relationships. In addition, Pearson’s correlation coefficients demonstrated that 990 lncRNAs and 7436 mRNAs were possibly in *trans*-regulatory relationships. These co-expressed mRNAs were enriched in various developmentally-related biological processes, such as myocyte proliferation and differentiation, myoblast differentiation, and myoblast fusion. The number of transcripts (906 lncRNAs and 4422 mRNAs) differentially expressed across various stages declined with the progression of differentiation. Then, 4422 differentially expressed genes were assigned to four clusters according to *K*-means analysis. Genes in the K1 cluster likely play important roles in myoblast proliferation and those in the K4 cluster were likely associated with the initiation of myoblast differentiation, while genes in the K2 and K3 clusters were likely related to myoblast fusion. This study provides a catalog of chicken lncRNAs and mRNAs for further experimental investigations and facilitates a better understanding of skeletal muscle development.

## 1. Introduction

Skeletal muscle, which accounts for approximately 40% of the body weight of mammals, is an important tissue involved in the regulation of metabolism, locomotion, and strength [1]. A loss or reduction in skeletal muscle mass results in weakness and impaired mobility and, if severe enough, in increased morbidity and mortality [2,3]. In livestock production, skeletal muscle develops into meat, which is the primary terminal product for human consumption. Therefore, the study of muscle development in agriculturally-important species is essential to achievements of increased body weight and muscle mass.

The process of muscle fiber development is nearly completed during the prenatal stage. Some reports suggest that animals with greater number of muscle fibers of moderate size produce a higher quantity of meat [4,5]. The development of skeletal muscle depends on myogenesis, which is a multistep process starting with myoblast proliferation, followed by their exit from cell cycle, differentiation, alignment, and fusion to form multinucleated myotubes that will further differentiate into mature fibers. During myogenesis, the degree of myoblasts proliferation and differentiation largely determines how many muscle fibers are formed. Therefore, understanding the events occurring during myogenesis is essential to achieve a higher quantity of meat. So far, the genetic factors influencing this process have been extensively studied using skeletal muscle myoblasts as model system because of their capable proliferation and differentiation (for example, see [6,7] among many others). However, the mechanisms underlying myogenesis are still sophisticated, especially as noncoding RNAs are emerging as integral components of the gene regulatory network in myogenesis.

Long non-coding RNAs (lncRNA), which are transcripts normally longer than 200 bases without evident protein coding regions, have recently emerged as important novel regulators of skeletal muscle development. With the development of RNA sequencing technology, several lncRNAs in different species and tissues have been identified and characterized [8,9,10]. In chickens, Li and Liu have identified 1995 and 2597 lncRNA transcripts, and their work largely enriched chicken lncRNA annotation [11,12]. A series of lncRNAs, such as linc-MD1, lnc-Six1, linc-H19, lnc-133b, and linc-YY1, have been proven to have an important impact on skeletal myogenesis [13,14,15,16,17]. Although many lncRNAs have been identified in chickens, a large number remains undiscovered. The profiles of lncRNAs obtained from chicken myoblasts and fused myotubes have not been reported, and lncRNAs involved in skeletal myogenesis are not yet elucidated.

The use of chicken embryonic myoblasts is a good model system [6,18] to understand how the genetic basis of myoblasts determines the outcome of the fate of myocytes and eventual muscle formation. Here, the expression profiles of lncRNAs and messenger RNAs (mRNAs) obtained from myoblasts and fused myotubes were investigated using RNA sequencing (RNA-seq) analysis. The features of the lncRNAs and mRNAs were characterized, and sets of differentially-expressed lncRNAs and mRNAs were identified by comparing the profiles of myoblasts at different stages of differentiation. The functions of differentially-expressed genes (DEGs) were annotated and the involved pathways were enriched. This investigation provides not only precious resources for chicken lncRNA studies, but also an approach to gain further insight into the complex biological processes to achieve a better understanding of the biology of myogenesis.

## 2. Materials and Methods

### 2.1. Ethics Statement

The study protocol was approved by the Animal Care Committee of the Department of Animal Science and Technology of Yangzhou University, Yangzhou, China (permit number: SYXK (Su) 2012-0029) and conducted in accordance with the guidelines of the Animal Use Committee of the Chinese Ministry of Agriculture (Beijing, China). All efforts were made to minimize animal suffering.

### 2.2. Chicken Primary Myoblast Isolation and Culture

Fertilized chicken eggs were obtained from Jiangsu Jinghai Livestock Industry Group (Haimen, China) and incubated at 37.8 °C under a relative humidity of 55–60%. The breast muscles of 12-day-old chicken embryos were collected, washed three times with Hank’s balanced salt solution (Solarbio, Beijing, China) formulated without magnesium and calcium, and then minced and digested with 0.2% collagenase type I (Sangon, Shanghai, China) in a water bath at 37 °C for 15 min. The digested cells were then dispersed by pipette and filtered to remove large debris using 200-, 400-, and 600-mesh screens (Solarbio). Cell were then collected by centrifugation at 350× *g* and resuspended in growth medium (GM), consisting of Dulbecco’s modified Eagle’s medium/F12 (1:1) (Sangon), supplemented with 20% fetal bovine serum (Invitrogen, Carlsbad, CA, USA), and 1% penicillin/streptomycin (Sangon). Enrichment of myoblasts cell population was accomplished by pre-plating the cells at 37 °C for 40 min on non-coated dish (Corning, New York, NY, USA) to remove the fibroblastic cells. This process was repeated two more times. To ensure uniformity of the samples, the purified myoblasts were mixed and seeded in twelve 60-mm dishes (Corning) at a density of 1 × 10^5^ cells/cm^2^. The culture dishes were incubated at 37 °C in a humidified atmosphere with 5% CO_2_ (Incubator: Binder, Tuttlingen, Germany). Myoblast differentiation was induced by replacing the 20% fetal bovine serum with 2% horse serum (Invitrogen). Cells were collected from the GM (100% confluency) and differentiation medium at 0, 24, 72, and 120 h (referred to as A0, A1, A3, and A5, respectively). Three biological replicates were collected at each of the four time points (n = 3).

### 2.3. Immunofluorescence

Primary myoblasts were fixed with 4% formaldehyde (Solarbio) for 30 min and then washed three times for 5 min each in phosphate-buffered saline (PBS) (Solarbio). The fixed cells were then permeabilized with 0.2% Triton X-100 (Solarbio) for 15 min and then blocked with normal goat serum (Solarbio) for 30 min at 37 °C. After blocking, the cells were incubated with anti-desmin (Bioss, Beijing, China) overnight at 4 °C. Afterward, the cells were then incubated with goat anti-rabbit lgG-Cy3 (Bioss) for 1 h at 37 °C. The cell nuclei were visualized using DAPI staining solution (Beyotime Institute of Biotechnology, Shanghai, China). 

### 2.4. RNA Isolation, Library Preparation, and Sequencing

Total RNA was isolated from each sample using TRIzol regent (Invitrogen, Carlsbad, CA, USA). The purity, concentration, and integrity of the RNA were checked using a NanoDrop 2000 spectrophotometer (Thermo Fisher Scientific, Waltham, MA, USA) and an Agilent 2100 Bioanalyzer (Agilent Technologies, Santa Clara, CA, USA), respectively. The RNA integrity number (RIN) of all samples was greater than 9.5.

Approximately 3 μg of RNA per sample were used to construct a complementary DNA (cDNA) library, according to the following procedures: the ribosome RNA (rRNA) was removed and strand-specific RNA-seq libraries were then generated using rRNA-depleted RNA. Briefly, after RNA fragmentation, double-stranded cDNA was synthesized by replacing dTTPs (deoxythymidine triphosphate) with dUTPs (deoxyuridine triphosphate) in reaction buffer used for second strand cDNA synthesis. The resulting double-stranded cDNA was ligated to adaptors, after being end-repaired and A-tailed. Single-strand cDNA was then obtained using USER (Uracil-Specific Excision Reagent) Enzyme (NEB, Ipswich, UK). Finally, PCR amplification was performed to enrich the cDNA libraries. Sequencing was performed on an Illumina Hiseq 2500 instrument using the TruSeq Cluster Kit v3-cBot-HS (Illumina, San Diego, CA, USA) to generate 150-bp paired-end reads.

### 2.5. Quality Control

The raw data were subjected to quality control using the Quality Control tool for High Throughput Sequence Data FastQC v0.11.2 [19]. The Phred score (Q20, Q30) and G + C content of the raw data were analyzed. At the same time, clean data were obtained by discarding reads containing adapter or ploy-N and low-quality reads (>50% of base with Phred scores <5) from the raw data.

### 2.6. Sequencing Data Analysis and Transcriptome Assembly

All the following analysis were based on the clean data with high quality. Reference genome and gene model annotation files were downloaded from following genome website (ftp://ftp.ensembl.org/pub/release-83/fasta/gallus_gallus/dna/). An index of the reference genome was built using Bowtie v2.0.6 [20] and paired-end reads were aligned to the chicken genome (Galgal 4.0) using TopHat v2.0.9 [21]. Mapped reads belonging to each sample were assembled with the Cufflinks v2.1.1 in a reference-based approach [22]. Transcripts from all samples were then merged together with the Cuffmerge tool to construct a consensus set of transcripts across the samples.

### 2.7. Identification of lncRNAs

Transcripts were filtered out from 12 chicken primary myoblast samples pooled by Cuffmerge using an in-house computational pipeline, which selects transcripts as candidate lncRNAs according to size (>200 bp), number of exons ≥ 2, expression levels (fragments per kilobase of exons per million mapped fragments, FPKM ≥ 5 overlaps of coding regions with no overlap with a known gene set), overlap of noncoding regions (no overlap with known pseudogenes, pre-microRNA, transfer RNA (tRNA), rRNA, or small nucleolar RNAs (snoRNAs)), and noncoding potential (CNCI [23] and CPC [24] scores > 0, Pfam-scan E-value > 0.001 [25], and PhyloCSF score > −20 [26]). Transcripts that passed through all of the filters mentioned above were collected as a candidate set of lncRNAs and were then blasted against the chicken lncRNAs retrieved from the Domestic-Animal Long Noncoding RNA Database (ALDB) v1.0 [27].

### 2.8. lncRNAs Targets Prediction and Annotation

The *cis* role of lncRNAs is to act on adjacent target genes, while the *trans* role is to identify every other lncRNA at the expression level. In the present study, protein-coding genes located ~10 kb upstream and downstream of the expressed lncRNAs were classified as lncRNAs of *cis*-target genes. Furthermore, the expression levels of the identified lncRNAs and the known coding genes from four different developmental stages were used to analyze the co-expression of lncRNAs and coding genes. The correlations between the expression levels of the lncRNAs and coding genes were evaluated using the Pearson’s correlation coefficient (*r* > 0.95 or < −0.95 and *p* < 0.05).

### 2.9. Screening and Clustering Analysis of Differentially Expressed lncRNAs and mRNAs

mRNA and lncRNA expression levels were calculated in fragments per kilobase of exons per million mapped fragments (FPKMs) using the Cuffdiff algorithm v2.1.1 (http://cole-trapnell-lab.github.io/cufflinks/cuffdiff/index.html). For biological replicates, a *p*-adjust value of >0.05 and an absolute log_2_ value (fold change) of <1 were considered to indicate a significant difference in mRNA expression. The DEGs were subjected to *K*-means clustering by the Euclidean distance method associated with complete linkage using the BMKCloud platform [28].

### 2.10. Gene Ontology and KEGG Enrichment Analysis

Gene Ontology (GO) and Kyoto Encyclopedia of Gene and Genomes (KEGG) functional annotation enrichment analysis were conducted using the DAVID database [29]. GO terms and KEGG pathways with *p*-values < 0.05 were considered significantly enriched.

### 2.11. Validation of Gene Expression by Quantitative Real-Time Polymerase Chain Reaction Analysis

Total RNA from the 12 samples used for the RNA-seq experiment was amplified by quantitative real-time polymerase chain reaction (qPCR). Single-strand cDNA was synthesized using the PrimeScriptTM RT Master Mix kit (TaKaRa Biotechnology, Dalian, China). qPCR was performed using an Applied Biosystems 7500 Real-Time PCR System (Life Technologies, Gaithersburg, MD, USA) with specific primers. qPCR amplifications were carried out in 20 µL reaction volumes containing 1 µL of cDNA, 10 µL of SYBR Premix Ex Taq polymerase (2×) (TaKaRa, Dalian, China), 0.4 µL of ROX Reference Dye II (50×) (TaKaRa), 0.4 µL of the forward primer (10 mmol/L), 0.4 µL of the reverse primer (10 mmol/L), and 6.8 µL of dH_2_O. The amplification started with initial denaturation step at 95 °C for 30 s followed by 40 cycles at 95 °C for 5 s and an annealing step at 60 °C for 34 s, at which point fluorescence was acquired. Finally, a dissociation curve to test PCR specificity was generated by one cycle at 95 °C for 15 s followed by 60 °C for 1 min and ramped to 95 °C with acquired fluorescence. Specific primers (Table S1) were designed based on sequences retrieved from the National Center for Biotechnology Information (NCBI) database. Gene expression levels were normalized to those of *HSP70* and *β-actin* to attain relative expression levels using the 2^−ΔΔCt^ method. Correlations between the RNA-seq and qPCR results were determined using the Spearman’s rank correlation coefficient.

## 3. Results

### 3.1. Proliferation and Differentiation of Embryonic Primary Myoblasts

**Figure 1:**
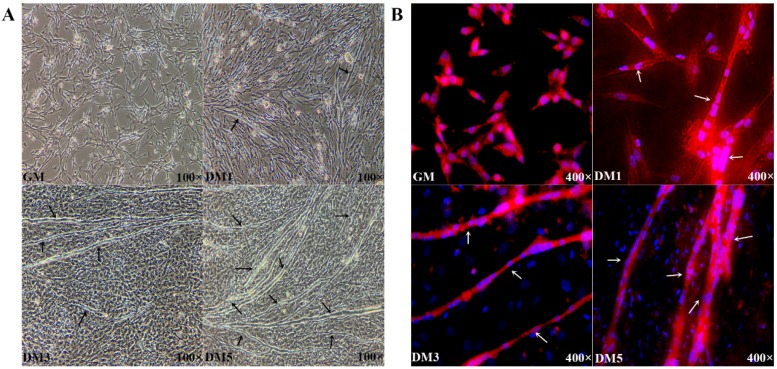
(**A**) Microscopic images of chicken primary myoblasts cultured in growth medium (GM) (100% confluence) or in differentiation medium (DM) for 24 h (DM1), 72 h (DM3) and 120 h (DM5). Black arrows represent myotubes; and (**B**) cells were fixed and immunostained for desmin in GM and DM. Nuclei were stained with DAPI. White arrows represent fused myotubes. Cells at different developmental stages were collected for use in the following procedures. The red and blue color represented cytoplasm and cell nucleus, respectively.

Chicken myoblasts were derived from primary cultures of embryonic breast muscle tissues and fused myotubes were generated by culturing the myoblasts in differentiation medium for 24, 72, or 120 h (Figure 1). The morphologies of the myoblasts and fused myotubes were observed under an inverted microscope, which revealed the formation of a few, small myotubes within 24 h after induction of differentiation. Immunofluorescence staining showed that the mononucleated myoblasts were fused to one other and the formation of elongated, multinucleated myotubes had markedly increased over the 120-h period. Cells at different developmental stages were collected for use in the following procedures.

### 3.2. Overview of RNA-Sequencing

Observation of the proliferation and differentiation of myoblasts to mature myotubes in vitro is a valuable tool for the characterization of cellular events during myogenesis. Therefore, the differentiation of separated chicken primary myoblasts was stimulated at specific time points and observed over the course of about five days. The Illumina Hiseq 2500 platform was used to perform RNA-seq for the 12 cDNA libraries, which resulted in the generation of more than 90 million raw reads from each library (Table S2). After quality control, most of the clean reads still comprised more than 90% of the raw data, with the exception of A0-2 (89.98%). More than 74.86% of the clean reads were perfectly mapped to the reference chicken genome (release: gaGal4). The uniquely-mapped reads ranged from 64.38% to 67.29% of clean reads (Table S2).

### 3.3. Identification and Characterization of lncRNAs in Chicken Myoblasts

**Figure 2:**
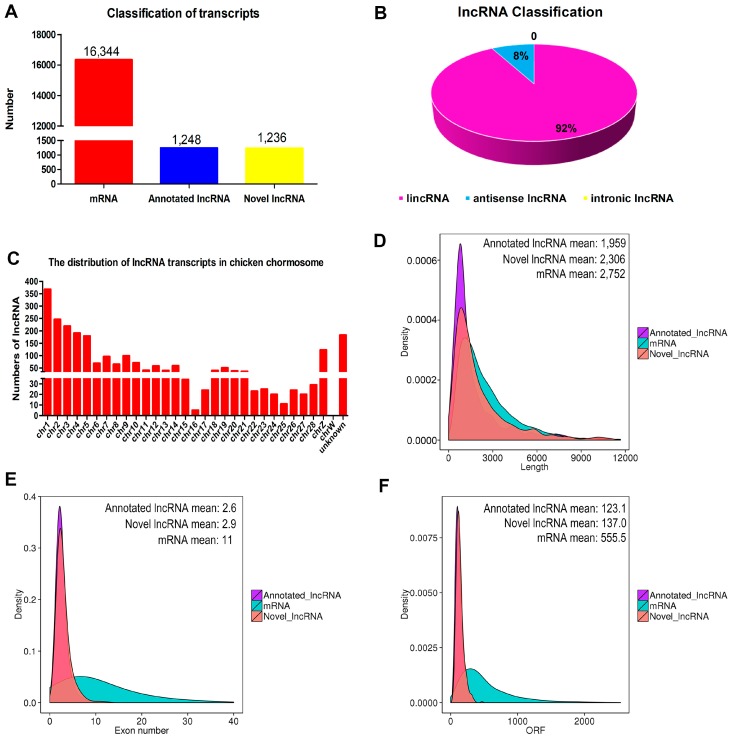
Characterization analysis of long non-coding RNAs (lncRNAs). (**A**) The classification of transcripts by RNA sequencing (RNA-seq); (**B**) lncRNA classification; (**C**) chromosome distribution of lncRNA transcripts in chicken primary myoblasts; (**D**) the length comparative analysis of lncRNAs and messenger RNA (mRNAs); (**E**) the exon number distribution of lncRNAs and mRNAs; and (**F**) the distribution of the open reading frame (ORF) length of lncRNAs and mRNAs.

Of a total of 18,828 transcripts that were identified, most (86.81%) were mRNAs (Figure 2A). Among all 2484 confirmed lncRNA transcripts, 1248 were previously annotated, and 1236 were novel (Figure 2A). Other than these, only two other types of lncRNAs were identified in the chicken primary myoblasts: an overwhelming majority of long intergenic non-coding RNAs (lincRNAs) (92.0%) and a minority of antisense lncRNAs (8.0%) (Figure 2B). The 2484 lncRNAs were distributed among chicken chromosomes 1–28 and Z. Quite notably, 367 lncRNAs were located on chromosome 1 (Figure 2C). The ggplot2 package [30] in the R environment was used to compare the genomic features of lncRNA to that of mRNA transcripts, as shown in Figure 2D–F. The average length of mRNA transcripts was 2752 bp with an average of 11 exons, which was larger than size of the lncRNA transcripts. Furthermore, the identified lncRNAs tended to have shorter open reading frames (ORF) than mRNAs (555.5 on average).

### 3.4. Functional Enrichment Analysis: GO and KEGG

**Figure 3:**
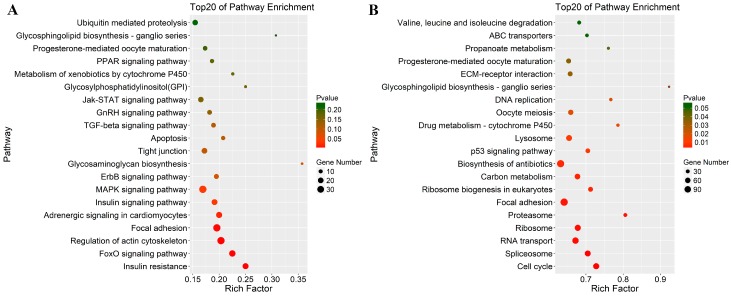
Kyoto Encyclopedia of Gene and Genomes (KEGG) enrichment of lncRNA-targets genes using DAVID software. (**A**) KEGG annotation for neighbor gene function of predicted lncRNAs; and (**B**) statistical KEGG enrichment for co-expressed gene function of identified lncRNAs.

To investigate the possible functions of lncRNAs in chicken myoblasts, the *cis*-targets of all lncRNAs were predicted using a threshold of 10-kb. As shown in Table S3, 1530 lncRNAs were transcribed close to 2041 protein-coding neighbors. GO analysis was then performed to identify the functions of the *cis* lncRNA targets, which showed that the highest number of genes were related to cell signaling and skeletal muscle contraction processes, such as cation binding (GO:0043169), metal ion binding (GO:0046872), and ion binding (GO:0043167) in addition to the other significantly enriched GO terms (*p* < 0.05), including protein modification process (GO:0036211), cellular protein modification process (GO:0006464), and regulation of molecular function (GO:0006509), suggesting that one of the principal roles of lncRNAs may be the mediation of protein modifications to regulate the expression of adjacent genes (Table S4). Pathway analysis indicated that seven pathways were significantly enriched (*p* < 0.05), including several related to muscle development via regulation of the cytoskeleton, insulin signaling pathway, MAPK signaling pathway, FoxO signaling, and focal adhesion (Figure 3A; Table S5). Taken together, the *cis* results showed that the protein-coding genes were regulated by neighboring lncRNAs involved in muscle development.

Then, the potential targets of lncRNAs in *trans*-regulatory relationship were predicted using co-expression analysis. A total of 105,926 interaction relationships (78,202 positively, and 27,724 negatively correlated) were detected between 990 lncRNAs and 7436 protein-coding genes in the reference chicken genome (Table S3). Functional analysis showed that the co-expressed genes were significantly enriched in 606 terms (451 under biological process, 83 under cellular component, and 72 under molecular function) that encompassed a variety of biological processes (Table S6). Importantly, some of the terms were related to muscle development, including muscle cell proliferation (GO: 0033002), muscle cell differentiation (GO: 0042692), myoblast fusion (GO: 0007520), myoblast differentiation (GO: 0045445), myotube differentiation (GO: 0014902), muscle cell development (GO: 0055001), striated muscle cell proliferation (GO: 0014855), and skeletal muscle cell differentiation (GO: 0035914). An additional 18 pathways were significantly enriched (*p* < 0.05), some of which were closely related to mitosis and cellular activities, such as the cell cycle, focal adhesion, the p53 signaling pathway, and extracellular matrix-receptor interactions (Figure 3B; Table S5). Overall, lncRNAs were involved in the *trans*-regulation of protein-coding genes associated with muscle development.

### 3.5. Differentially-Expressed lncRNAs and mRNAs during Primary Myoblast Differentiation

**Figure 4:**
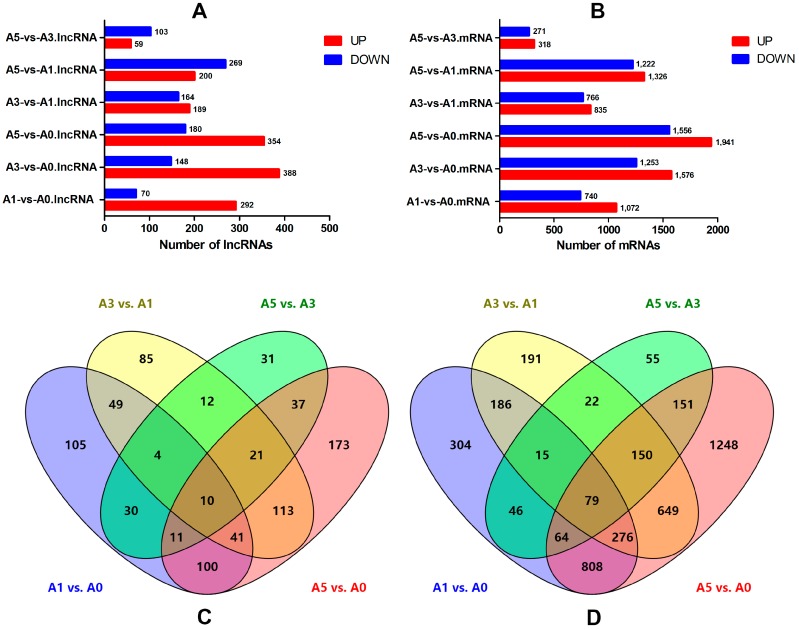
Differentially-expressed lncRNA and mRNA in chicken primary myoblasts at four differentiation stages. (**A**) The number of up-regulated and down-regulated lncRNAs; (**B**) the number of up-regulated and down-regulated mRNAs; (**C**) a Venn diagram of differentially-expressed lncRNAs in four comparisons (A1 vs. A0, A3 vs. A1, A5 vs. A3 and A5 vs. A0); and (**D**) a Venn diagram of differentially-expressed mRNAs in four comparisons (A1 vs. A0, A3 vs. A1, A5 vs. A3, and A5 vs. A0).

To investigate the key lncRNAs and mRNAs involved in myoblast differentiation, RNA-seq was performed to detect the differentially-expressed lncRNAs (DE-lncRNAs) and genes (DEGs) at four developmental stages of chicken primary myoblasts in vitro (A0, A1, A3, and A5). DE-lncRNAs and DEGs were identified using Cuffdiff analysis (|(fold change)| > 2; *p*-adjust < 0.05). Lastly, 906 lncRNAs and 4422 mRNAs were found to be differentially expressed during myoblast differentiation (Figure 4A,B and Table S7). The number of up-regulated mRNAs was higher than the number of down-regulated mRNAs during myoblast development, while the lncRNAs shared a common feature, with the exception of A5 vs. A3 and A5 vs. A1. To further analyze the interactions among the differentially expressed lncRNAs, 362, 353, 162, and 534 differentially-expressed lncRNAs were used in four comparisons (A1 vs. A0, A3 vs. A1, A5 vs. A3, and A5 vs. A0, respectively) to construct a Venn diagram. Meanwhile, 1812, 1601, 589, and 3497 differentially-expressed mRNAs in the aforementioned four comparisons were used to construct a Venn diagram. As shown in Figure 4C,D, 89 differentially-expressed genes (10 lncRNAs and 79 mRNAs) were common among the four comparisons.

**Figure 5:**
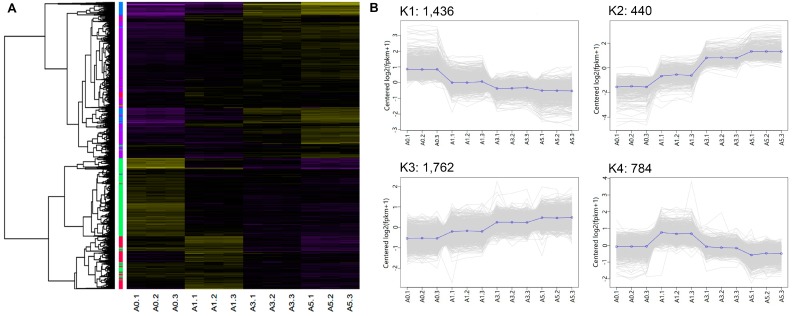
Hierarchical clustering of differentially-expressed genes (DEGs) (4422). (**A**) Heatmap plot of DEGs using the hierarchical clustering method; four cluster are shown; decreased (**purple**) and increased (**yellow**) expression of DEGs are distinguished from different stages; and (**B**) expression patterns of genes in the four clusters, namely K1–K4, corresponding to the hierarchical heatmap. The blue line represented the centered expression pattern of genes in each cluster.

To extract additional biological information from the multi-dimensional transcriptome dataset, clustering analysis we performed using the *K*-means method to identify co-expression in DEG clusters. Four main clusters were plotted with the expression patterns of the involved genes (Figure 5, Table S8). The K1 cluster included 1436 genes with an overall decreasing trend among most of these genes. Notably, most DEGs in the K4 cluster had the highest expression levels during the initial differentiation stage. Additionally, the DEGs in the K2 and K3 clusters had some similar expression patterns, which showed an overall upregulated trend during myofibrillogenesis. Dynamic changes in gene expression reflect an intrinsic mechanism of how an organism responds to developmental and environmental signals. That is, the DEGs with different expression patterns might play different and necessary roles in myoblast proliferation and differentiation. Therefore, to better understand their roles and relevance in muscle development, GO analysis was performed to identify enriched (*p* < 0.05) biological process terms for each cluster (Table S9). The results showed that DEGs in the K1 cluster were mainly involved in the cell cycle, DNA replication, and mitosis, whereas the functions of DEGs in the K4 cluster were mainly involved in muscle development and muscle cell differentiation. Based on these findings, one can infer that DEGs in the K1 cluster might play important roles in myoblast proliferation, whereas those in the K4 cluster might play vital roles in the initial steps of myoblast differentiation. Interestingly, there was a great deal of similarity in DEG function in the K2 and K3 clusters, which shared 75 overlapping GO terms. This phenomenon may have resulted from the similarities in expression patterns. Cell adhesion and migration are the basis for the fusion of myoblasts. Genes involved in cell adhesion and migration were greatly enriched in these two clusters. Therefore, we predicted that the DEGs in the K2 and K3 clusters might play essential roles in myoblast fusion and myotube formation. The overrepresented GO terms of DEGs involved in biological processes are shown in Table 1.

### 3.6. Verification of Gene Expression Profiles Using qPCR

**Figure 6:**
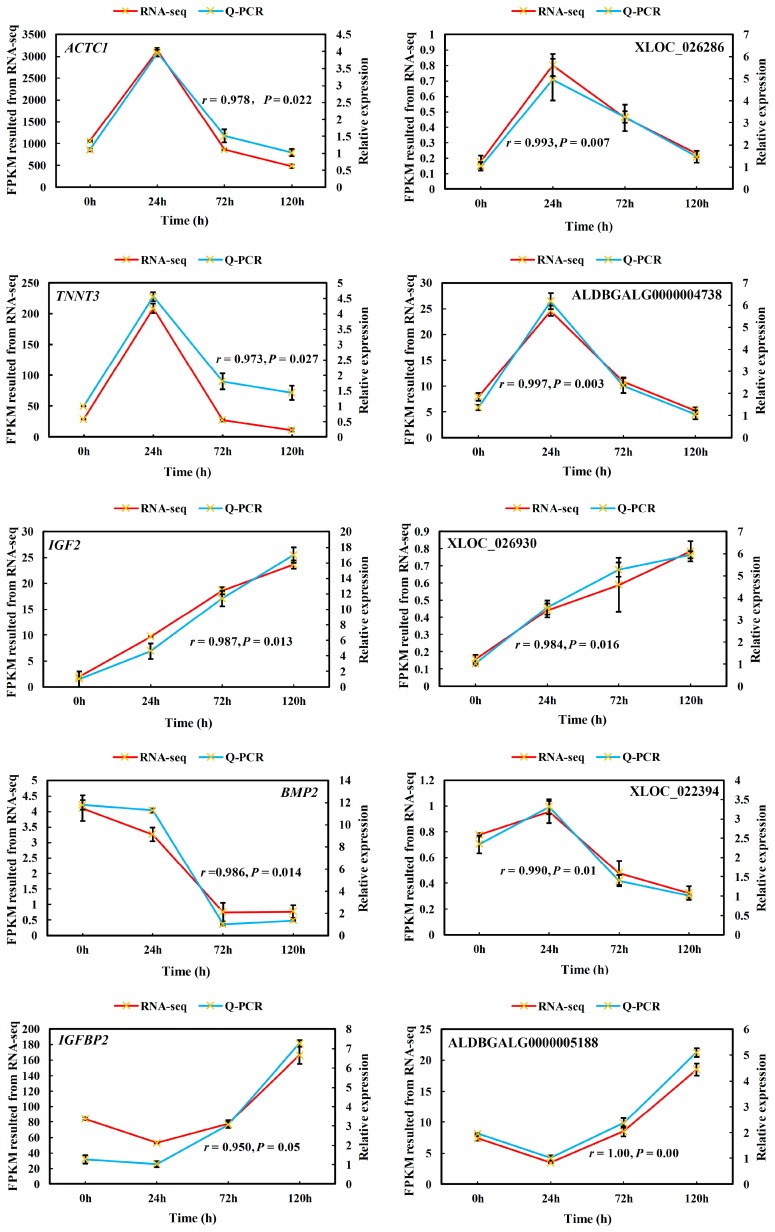
qPCR validation of differentially-expressed lncRNAs and their cis-target genes. FPKM: fragments per kilobase of exons per million mapped fragments.

To confirm the accuracy and reproducibility of the RNA-seq results, five well-known DE-lncRNA target genes that affect muscle development, including *ACTC1*, *TNNT3*, *IGF2*, *BMP2*, and *IGFBP2*, and their corresponding lncRNA regulators XLOC_026286, ALDBGALG0000004738, XLOC_026930, XLOC_022394, and ALDBGALG0000005188, were chosen for qPCR validation. The results showed that the expression patterns of all five lncRNAs and their *cis*-target genes were in excellent agreement with the RNA-seq findings (Figure 6), indicating the reliability of our RNA-seq data.

## 4. Discussion

Skeletal muscle growth and development consists of a series of exquisitely regulated changes in gene expression from the embryo to adult stages. Deciphering these developmental changes in agriculturally-important species is essential to the production of high-quality meat products. One of the greatest surprises was that most transcription originated from the noncoding regions of the genome [31]. The existence of non-coding RNAs (ncRNAs), including small ncRNAs (such as piwi-interacting RNA (piRNA), small interfering RNA (siRNA), and microRNA (miRNA)) and lncRNAs revealed the complexity of genome expression. However, the roles of small ncRNAs have been relatively well characterized in skeletal muscle biology [32,33]. In contrast, lncRNAs were identified only recently and we are still in the infancy of studying this novel class of ncRNAs in skeletal muscle. Despite the fact that transcriptomic research on chicken lncRNA have been carried out, transcriptome analysis of chicken muscle is performed only at the embryonic or adult stage [11,34]. In comparison to the complexity of muscle tissue at different developmental stages in vivo, embryonic myoblasts in vitro provide an easier method to understand the cellular events occurring during skeletal myogenesis. In the present study, the Illumina Hiseq 2500 platform was used to determine the identity and expression level of lncRNAs and mRNAs involved in muscle formation in an in vitro cell culture system, where myoblasts were allowed to fuse to multinucleated myotubes. To the best of our knowledge, this study is the first to screen and sequence lncRNAs and mRNAs involved in the regulation of the proliferation and differentiation of chicken myoblasts. As a result, an average of 14.43 Gb of clean bases were obtained from myoblasts and fused myotubes. Most of the 1154 million clean reads obtained (77.8%) were perfectly mapped to the current Galga 4.0 genome assembly, which was higher than the percentage obtained for the transcriptomes of sperm (73.1%) [12] and leg muscle (73.4%) [35]. The extensive sequencing depth contributed to the very high sequence coverage of the reference chicken genome. With the number sequencing depth and higher mapping ratio, more transcripts were obtained.

Due to the rigorous screening criteria, a total of 2484 high-confidence lncRNAs were identified, of which 2288 were lncRNAs and 198 were antisense lncRNAs. Furthermore, only 14% of all lncRNAs were mapped with reported chicken lncRNAs from the ALDB database (8923), which was consistent with the results of previous reports [36,37]. A possible reason for this difference is that chicken lncRNAs tend to be highly tissue-specific. Moreover, the lncRNAs transcripts were widespread in chromosomes, including 28 autosomes and the Z-chromosome, which at least reflected the complexity and functional diversity of these lncRNAs. Notably, the proportion of lncRNAs located in chromosome 1 was the highest compared with other chromosomes, which was similar to a previous study of chicken leg muscle [11]. Many reports have revealed that lncRNAs share many common characteristics among species, including shorter transcript and open reading frame lengths, and fewer exons, as compared to mRNAs [38,39]. These features were also observed in this study, indicating the reliability of lncRNA identification. 

Recent studies have demonstrated that the functions of lncRNAs can be inferred from adjoining or co-expressed protein-coding genes [40,41,42]. In the *cis* prediction, the protein products of the neighboring genes of all identified lncRNAs were used to predict their possible roles during myogenesis. Consequently, functional enrichment of the neighboring coding genes revealed that major enriched pathways were associated with muscle development. Combined with differential expression analysis, many of the lncRNAs and their target genes were differentially expressed, including some potentially involved in myogenesis regulation. Of these, lncRNA XLOC_026286 was predicted to act on the target gene *ACTC1*, a predominant striated α-actin isoform that is expressed in the fetal skeletal muscle and adult heart [43,44]. Some reports have demonstrated that *ACTC1* over-expression in postnatal skeletal muscle could effectively rescue *ACTA1*-related diseases [45,46], congenital myopathies caused by mutations in *ACTA1* [47]. lncRNA ALDBGALA0000004738 was predicted to act on the potential target gene *TNNT3*, a skeletal muscle contractile gene that has been reported to be associated with distal arthrogryposis syndromes [48]. lncRNA XLOC_026930 was predicted to act on the neighboring gene *IGF2*, an embryonic regulator of myogenesis and an autocrine factor that initiates myoblast differentiation in vitro [49,50]. lncRNA XLOC_022394 was predicted to act on the target gene *BMP2*, a member of the transforming growth factor β super family. In this study, the *BMP2* gene showed a notable overall trend of downregulation, suggesting its negative roles in myoblast differentiation. This is in accordance with a previous notion that *BMP2* functions as a negative regulator in the differentiation of human myoblasts [51]. These findings suggest that lncRNAs might regulate myogenesis and muscle regeneration through actions on neighboring genes. However, these predicted lncRNA-mRNA pairs require further experimental verification. 

Still, many lncRNAs were found to also function in *trans* mode to target gene loci distant from the transcription sites of the lncRNAs [33]. Co-expression analysis identified 990 lncRNAs that were related to 7436 coding genes based on the expression correlation coefficient (r) of >0.95 or <0.95. Functional enrichment analysis of the co-expressed genes was mainly involved in the cell cycle, mitosis, and cellular activities that are closely related to myofibrillogenesis. Furthermore, co-expression analysis showed that a cluster of lncRNAs often targeted coding genes that were specifically expressed in muscle and were involved in muscle development (e.g., *MyoD*, *MyoG*, *Myf5*, *MEF2C*, *TNNT3*, *GDF8*, and *TMEM8C*). Combined with differential expression analysis, DEGs of interest were selected to perform a reverse lookup of co-expressed lncRNAs to help refine the most likely lncRNA-mRNAs pairs for subsequent experimental validation. For example, 16 DE-lncRNAs were co-expressed with *TMEM8C*, the muscle-specific protein known to be absolutely essential for fusion, development, and regeneration of myoblasts in mammals [52,53]. We can forecast boldly that the 16 DE-lncRNAs might play the same important roles as *TMEM8C* in skeletal muscle development. In addition, 26 DE-lncRNAs were reverse-expressed with *FAK*, a key mediator of integrin signaling, which has been implicated in the regulation of cell migration and cell cycle procession [54]. Liu et al. reported that *FAK* is a negative regulator during the processes of myoblast migration and fusion [55]. This interesting observation indicates the diverse and complex functions of lncRNAs and deserves further investigation. Therefore, our ongoing efforts will focus on the function of those most likely lncRNAs to provide more fundamental information to further elucidate their regulatory mechanisms in skeletal muscle development at the molecular level.

Massive RNA-seq experiments comprising two or more time points that correspond to the development or distinct treatment are useful to unravel the intrinsic and dynamic regulatory mechanisms [56]. For genes with an explicit expression curve from a specific cluster, their similar expression trend can be a basis for the postulation that these genes share a common responsive behavior to a developmental or environmental stimulus [57,58]. Therefore, in this study, four clusters were defined according to the *K*-means results to further illustrate the relationships between DEGs with various expression patterns. GO analysis was performed for each co-expression gene cluster, which showed that the stage-specific clusters were enriched in terms of the expected functional gene sets. The K1 cluster exhibited a developmental trend with the highest expression during the proliferation stage, followed by a progressive decline when the cells underwent differentiation. As expected, these genes were enriched in the functional categories related to cell proliferation, such as the cell cycle, DNA replication, and mitosis. A group of DEGs were reportedly related to myoblast proliferation, which include *YY1* [59], *PAX3* [60], *E2F3* [61], *SIX1* [62], and *MYF5* [63], among others. By contrast, the K2 and K3 clusters exhibited changes in the opposite direction, with an overall upregulated trend. Myoblast fusion is an important step in the formation of multinucleated fibers. Cell migration and adhesion are essential for cell-cell fusion of myoblasts. As we can imagine, the functional categories of the two clusters were mainly involved in cell migration, adhesion, and cell-extracellular interactions, suggesting possible roles over the entire myofibrillogenesis process. For example, *IGF2* [64], *FABP4* [65], *KLF4* [66], *FAK* [67], and *RAC2* [68] were recently reported to be involved in cell migration and adhesion. Notably, genes in the K4 cluster showed significant and specific up-regulation at day 1 of differentiation, indicating the importance of the contribution of these genes to the initiation of myoblast differentiation. Without question, these genes were mainly enriched in terms related to muscle development and muscle differentiation. Members of this cluster, including *MYOG*, *MEF2C*, *MEF2D*, and *TMEM8C* [69], reportedly regulate myoblast differentiation. In fact, the fusion of myoblasts denotes that mononucleated myoblasts exit the cell cycle, begin differentiation, and fuse with one another to generate syncytial myofibers [52]. The functional categories of each stage-specific cluster are in accordance with the process of myogenesis. Moreover, this method might be a good choice for the discovery of putative genes as candidates for further experimental validation.

Furthermore, the accuracy of RNA sequencing was confirmed by qPCR in this study. Of the five lncRNA-mRNA pairs selected for testing, the results were consistent with the RNA-seq data, thereby confirming the reliability of RNA-seq and laying a solid foundation for further exploration.

## 5. Conclusions

In summary, the application of transcriptome analysis during myoblast proliferation and differentiation provided a catalog of lncRNAs and mRNAs to further understand the regulatory roles of these RNA molecules in chicken muscle development. In future studies, we plan to investigate the functions of some lncRNAs to further elucidate the regulatory mechanisms underlying chicken muscle development. Perhaps, the lncRNAs identified in this study can be utilized to improve the meat production in livestock.

## Figures and Tables

**Table 1:** Overrepresented GO terms of DEGs involved in different clusters.

Cluster (# of genes)	Example genes	Enriched GO terms	# of gens	*p*-value
K1 (1,436)	YY1, TGFβ1, SOX8, SIX1, SIX2, PAX3, PAX7, MYF5, MYF6, ID1, ID2, E2F7, E2F3, E2F1, E2F2, CDC7, CDC6, CDC45, CDC20, CDK1, CDK2, CDK6, CDK7	Cell cycle process	138	3.81 × 10^−22^
		DNA metabolic process	123	5.17 × 10^−22^
		Cell cycle	156	9.67 × 10^−22^
		Chromosome organization	132	4.51 × 10^−21^
		DNA replication	59	2.94 × 10^−20^
		Mitotic cell cycle process	92	1.86 × 10^−17^
		Nuclear division	73	5.56 × 10^−17^
		DNA repair	76	8.60 × 10^−17^
		Mitotic cell cycle	98	3.19 × 10^−16^
		Chromosome segregation	54	2.18 × 10^−15^
K2 (440)	MYOC, IGF2, MYO1D, FGF7, FGF18, FABP4, ECM2, DCX, FAP, FAT4, KLF15, LBP, SHOX, TF	Extracellular structure organization	18	5.03 × 10^−7^
		Extracellular matrix organization	18	5.03 × 10^−7^
		Chemotaxis	24	1.0 6 ×10^−5^
		Taxis	24	1.13 × 10^−5^
		Cellular response to chemical stimulus	59	2.49 × 10^−5^
		Response to external stimulus	52	3.50 × 10^−5^
		Regulation of cell migration	27	6.87 × 10^−5^
		Cellular response to organic substance	49	7.19 × 10^−5^
		Cell adhesion	41	9.90 × 10^−5^
		Biological adhesion	41	1.09 × 10^−4^
K3 (1,762)	FLI1, KLF4, WNT9A, CTR, WNT5A, EGFR, BMP5, FAK, FGF14, FGF9, RAC2, MYH6, MYH10, MYCN, MXD4, IGFBP2, IGFBP4, IGFBP5	Locomotion	139	6.05 × 10^−8^
		Movement of cell or subcellular component	148	1.79 × 10^−6^
		Enzyme linked receptor protein signaling pathway	86	3.16 × 10^−6^
		Transmembrane receptor protein tyrosine kinase signal	61	7.29 × 10^−6^
		Organ morphogenesis	93	7.34 × 10^−6^
		Biological adhesion	118	9.30 × 10^−6^
		Skeletal system development	59	1.04 × 10^−5^
		Cell adhesion	117	1.25 × 10^−5^
		Skeletal system morphogenesis	36	1.42 × 10^−5^
		Neurogenesis	127	1.59 × 10^−5^
K4 (784)	TNNT3, SRF, RGS2, Myosin, MYOG, TMEM8C, DLL1 NOTCH1, MYOD, MEF2C, GDF8, MEF2D, MAPK13, DIK2, BMP4, β-FGF, ACTC1, MTOR, SMYD1	Muscle structure development	61	3.24 × 10^−20^
		Muscle system process	41	6.97 × 10^−18^
		Muscle contraction	37	3.07 × 10^−17^
		Striated muscle cell differentiation	35	2.35 × 10^−15^
		System process	87	5.03 × 10^−14^
		Muscle cell differentiation	41	1.18 × 10^−13^
		Muscle tissue development	41	2.72 × 10^−13^
		Muscle organ development	36	1.40 × 10^−12^
		Striated muscle tissue development	38	1.51 × 10^−12^
		Muscle cell development	26	3.83 × 10^−12^

## Supplementary Materials

The following are available online at www.mdpi.com/2073-4425/9/1/34/s1. Table S1. Primers for qPCR; Table S2. Summary of draft reads of 12 libraries by RNA-sequencing; Table S3. lncRNA targets predicted by *cis*- and *trans*-acting; Table S4. Functional enrichment analysis of protein-coding genes targeted by *cis*-acting lncRNAs; Table S5. KEGG analysis of protein-coding genes targeted by *cis*-and *trans*-acting lncRNAs; Table S6. Functional enrichment analysis of protein-coding genes co-expressed with lncRNAs; Table S7. List of differentially-expressed lncRNAs and mRNAs from four myoblasts developmental stages; Table S8. Differentially-expressed genes for *K*-means analysis; Table S9. Functional enrichment analysis of DEGs involved in K1, K2, K3, and K4 clusters.

## References

[1] Frontera W.R., Ochala J. (2015). Skeletal muscle: A brief review of structure and function. Calcif. Tissue Int..

[2] Coelen R.J., Wiggers J.K., Nio C.Y., Besselink M.G., Busch O.R., Gouma D.J., van Gulik T.M. (2015). Preoperative computed tomography assessment of skeletal muscle mass is valuable in predicting outcomes following hepatectomy for perihilar cholangiocarcinoma. HPB.

[3] Szulc P., Munoz F., Marchand F., Chapurlat R., Delmas P.D. (2010). Rapid loss of appendicular skeletal muscle mass is associated with higher all-cause mortality in older men: The prospective MINOS study. Am. J. Clin. Nutr..

[4] Koomkrong N., Theerawatanasirikul S., Boonkaewwan C., Jaturasitha S., Kayan A. (2015). Breed-related number and size of muscle fibres and their response to carcass quality in chickens. Ital. J. Anim. Sci..

[5] Rehfeldt C., Fiedler I., te Pas M.F.W., Everts M.E., Haagaman H.P. (2004). Number and size of muscle fibres in relation to meat production. Muscle Development of Livestock Animals: Physiology, Genetic, and Meat Quality.

[6] Sato F., Kurokawa M., Yamauchi N., Hattori M.A. (2006). Gene silencing of myostatin in differentiation of chicken embryonic myoblasts by small interfering RNA. Am. J. Physiol.-Cell Physiol..

[7] Yi X., Tao Y., Lin X., Dai Y., Yang T., Yue X., Jiang X., Li X., Jiang D.S., Andrade K.C. (2017). Histone methyltransferase Setd2 is critical for the proliferation and differentiation of myoblasts. Biochim. Biophys. Acta.

[8] Billerey C., Boussaha M., Esquerré D., Rebours E., Djari A., Meersseman C., Klopp C., Gautheret D., Rocha D. (2014). Identification of large intergenic non-coding RNAs in bovine muscle using next-generation transcriptomic sequencing. BMC Genom..

[9] Gao P.F., Guo X.H., Du M., Cao G.Q., Yang Q.C., Pu Z.D., Wang Z.Y., Zhang Q., Li M., Jin Y.S. (2017). LncRNA profiling of skeletal muscles in Large White pigs and Mashen pigs during development. J. Anim. Sci..

[10] Ren C., Deng M., Fan Y., Yang H., Zhang G., Feng X., Li F., Wang D., Wang F., Zhang Y. (2017). Genome-wide analysis reveals extensive changes in lncRNAs during skeletal muscle development in Hu sheep. Genes.

[11] Li Z., Ouyang H., Zheng M., Cai B., Han P., Abdalla B.A., Nie Q., Zhang X. (2016). Integrated analysis of long non-coding RNAs (lncRNAs) and mrna expression profiles reveals the potential role of lncRNAs in skeletal muscle development of the chicken. Front. Physiol..

[12] Liu Y., Sun Y., Li Y., Bai H., Xue F., Xu S., Xu H., Shi L., Yang N., Chen J. (2017). Analyses of long non-coding RNA and mRNA profiling using RNA sequencing in chicken testis with extreme sperm motility. Sci. Rep..

[13] Cai B., Li Z., Ma M., Wang Z., Han P., Abdalla B.A., Nie Q., Zhang X. (2017). LncRNA-Six1 encodes a micropeptide to activate Six1 in *cis* and is involved in cell proliferation and muscle growth. Front. Physiol..

[14] Cesana M., Cacchiarelli D., Legnini I., Santini T., Sthandier O., Chinappi M., Tramontano A., Bozzoni I. (2011). A long noncoding RNA controls muscle differentiation by functioning as a competing endogenous RNA. Cell.

[15] Cong F.J., Yan L., Xiang B.D., Xin L., Lin L.Z., Xin F.L., Hong G. (2017). Lnc133b, a novel, long non-coding RNA, regulates bovine skeletal muscle satellite cell proliferation and differentiation by mediating miR-133b. Gene.

[16] Sun K., Zhou L., Zhao Y., Wang H., Sun H. (2016). Genome-wide RNA-seq and ChiP-seq reveal Linc-YY1 function in regulating YY1/PRC2 activity during skeletal myogenesis. Genom. Data.

[17] Xu X., Ji S., Li W., Yi B., Li H., Zhang H., Ma W. (2017). LncRNA H19 promotes the differentiation of bovine skeletal muscle satellite cells by suppressing Sirt1/Foxo1. Cell. Mol. Biol. Lett..

[18] Luo W., Wu H., Ye Y., Li Z., Hao S., Kong L., Zheng X., Lin S., Nie Q., Zhang X. (2014). The transient expression of miR-203 and its inhibiting effects on skeletal muscle cell proliferation and differentiation. Cell Death Dis..

[19] Schmieder R., Edwards R. (2011). Quality control and preprocessing of metagenomic datasets. Bioinformatics.

[20] Langmead B., Salzberg S.L. (2012). Fast gapped-read alignment with Bowtie 2. Nat. Methods.

[21] Kim D., Pertea G., Trapnell C., Pimentel H., Kelley R., Salzberg S.L. (2013). Tophat2: Accurate alignment of transcriptomes in the presence of insertions, deletions and gene fusions. Genome Biol..

[22] Trapnell C., Williams B.A., Pertea G., Mortazavi A., Kwan G., Baren M.J., Salzberg S.L., Wold B.J., Pachter L. (2010). Transcript assembly and quantification by RNA-seq reveals unannotated transcripts and isoform switching during cell diffrentiation. Nat. Biotechnol..

[23] Sun L., Luo H., Bu D., Zhao G., Yu K., Zhang C., Liu Y., Chen R., Zhao Y. (2013). Utilizing sequence intrinsic composition to classify protein-coding and long non-coding transcripts. Nucleic Acids Res..

[24] Kong L., Zhang Y., Ye Z.Q., Liu X.Q., Zhao S.Q., Wei L., Gao G. (2007). CPC: Assess the protein-coding potential of transcripts using sequence features and support vector machine. Nucleic Acids Res..

[25] Bateman A., Birney E., Cerruti L., Durbin R., Etwiller L., Eddy S.R., Griffithsjones S., Howe K.L., Marshall M., Sonnhammer E.L. (2002). The Pfam protein families database. Nucleic Acids Res..

[26] Lin M.F., Irwin J., Manolis K. (2011). PhyloCSF: A comparative genomics method to distinguish protein coding and non-coding regions. Bioinformatics.

[27] Li A., Zhang J., Zhou Z., Wang L., Liu Y., Liu Y. (2015). ALDB: A domestic-animal long noncoding RNA database. PLoS ONE.

[28] Hsu C., Lin C., Ouyang M., Guo Y.K. (2013). Biocloud: Cloud computing for biological, genomics, and drug design. Biomed Res. Int..

[29] Huang D.W., Sherman B.T., Lempicki R.A. (2009). Syatematic and intergrative analysis of large gene lists using DAVID bioinformatics resources. Nat. Protoc..

[30] Ginestet C. (2011). Ggplot2: Elegant graphics for data analysis by H. Wickham. J. R. Stat. Soc..

[31] Lander E.S., Linton L.M., Birren B., Nusbaum C., Zody M.C., Baldwin J., Devon K., Dewar K., Doyle M., Fitzhugh W. (2001). Initial sequencing and analysis of the human genome. Nature.

[32] Luo W., Nie Q., Zhang X. (2013). MicroRNAs involved in skeletal muscle differentiation. J. Genet. Genom..

[33] Nie M., Deng Z.L., Liu J., Wang D.Z. (2015). Noncoding RNAs, emerging regulators of skeletal muscle development and diseases. BioMed Res. Int..

[34] Nie Q. Integration analysis of lncRNA regulatory network involving miRNA and mRNA in chicken breast muscle. Proceedings of the Plant and Animal Genome Asia.

[35] Xue Q., Zhang G., Li T., Ling J., Zhang X., Wang J. (2017). Transcriptomic profile of leg muscle during early growth in chicken. PLoS ONE.

[36] Shen Y., Mao H., Huang M., Chen L., Chen J., Cai Z., Ying W., Xu N. (2016). Long noncoding RNA and mRNA expression profiles in the thyroid gland of two phenotypically extreme pig breeds using Ribo-Zero RNA sequencing. Genes.

[37] Tsoi L.C., Lyer M.K., Stuart P.E., Swindell W.R., Gudjonsson J.E., Tejasvi T., Satkar M.K., Li B., Ding J., Voorhees J.J. (2015). Analysis of long non-coding RNAs highlights tissue-specific expression patterns and epigenetic profiles in normal and psoriatic skin. Genome Biol..

[38] Lu X., Chen X., Mu M., Wang J., Wang X., Wang D., Yin Z., Fan W., Wang S., Guo L. (2016). Genome-wide analysis of long noncoding RNAs and their responses to drought stress in cotton (*Gossypium hirsutum* L.). PLoS ONE.

[39] Wang Y., Xue S., Liu X., Liu H., Hu T., Qiu X., Zhang J., Lei M. (2016). Analyses of long non-coding RNA and mRNA profiling using RNA sequencing during the pre-implantation phases in pig endometrium. Sci. Rep..

[40] Zhan S., Dong Y., Zhao W., Guo J., Zhong T., Wang L., Li L., Zhang H. (2016). Genome-wide identification and characterization of long non-coding RNAs in developmental skeletal muscle of fetal goat. BMC Genom..

[41] Zhang S., Qin C., Cao G., Xin W., Feng C., Zhang W. (2016). Systematic analysis of long noncoding RNAs in the senescence-accelerated mouse prone 8 brain using RNA sequencing. Mol. Ther. Nucleic Acids.

[42] Zhou J., Xiong Q., Chen H., Yang C., Fan Y. (2017). Identification of the spinal expression profile of non-coding RNAs involved in neuropathic pain following spared nerve injury by sequence analysis. Front. Mol. Neurosci..

[43] Ilkovski B., Clement S., Sewry C., North K.N., Cooper S.T. (2005). Defining α-skeletal and α-cardiac actin expression in human heart and skeletal muscle explains the absence of cardiac involvement in ACTA1 nemaline myopathy. Neuromuscul. Disord..

[44] Vandekerckhove J., Bugaisky G., Buckingham M. (1986). Simultaneous expression of skeletal muscle and heart actin proteins in various striated muscle tissues and cells. A quantitative determination of the two actin isoforms. J. Biol. Chem..

[45] Nowak K.J., Ravenscroft G., Jackaman C., Filipovska A., Davies S.M., Lim E.M., Squire S.E., Potter A.C., Baker E., Clément S. (2009). Rescue of skeletal muscle α-actin–null mice by cardiac (fetal) α-actin. J. Cell Biol..

[46] Ravenscroft G., Mcnamara E., Griffiths L.M., Papadimitriou J.M., Hardeman E.C., Bakker A.J., Davies K.E., Laing N.G., Nowak K.J. (2013). Cardiac α-actin over-expression therapy in dominant ACTC1 disease. Hum. Mol. Genet..

[47] Nowak K.J., Ravenscroft G., Laing N.G. (2013). Skeletal muscle α-actin diseases (actinopathies): Pathology and mechanisms. Acta Neuropathol..

[48] Sung S.S., Brassington A.M., Krakowiak P.A., Carey J.C., Jorde L.B., Bamshad M. (2003). Mutations in *TNNT3* cause multiple congenital contractures: A second locus for distal arthrogryposis type 2B. Am. J. Hum. Genet..

[49] Florini J.R., Magri K.A., Ewton D.Z., James P.L., Grindstaff K., Rotwein P.S. (1991). ‘Spontaneous’ differentiation of skeletal myoblasts is dependent upon autocrine secretion of insulin-like growth factor-II. J. Biol. Chem..

[50] Ge Y., Sun Y., Chen J. (2011). IGF-II is regulated by microRNA-125b in skeletal myogenesis. J. Cell Biol..

[51] Aoyama K., Yamane A., Suga T., Suzuki E., Fukui T., Nakamura Y. (2011). Bone morphogenetic protein-2 functions as a negative regulator in the differentiation of myoblasts, but not as an inducer for the formations of cartilage and bone in mouse embryonic tongue. BMC Dev. Biol..

[52] Millay D.P., O’Rourke J.R., Sutherland L.B., Bezprozvannaya S., Shelton J.M., Basselduby R., Olson E.N. (2013). Myomaker is a membrane activator of myoblast fusion and muscle formation. Nature.

[53] Millay D.P., Sutherland L.B., Bassel-Duby R., Olson E.N. (2014). Myomaker is essential for muscle regeneration. Genes Dev..

[54] Shen T.L., Guan J.L. (2001). Differential regulation of cell migration and cell cycle progression by FAK complexes with Src, Pi3k, Grb7 and Grb2 in focal contacts. FEBS Lett..

[55] Liu L. (2014). Tyrosine kinases Abl and Fak inhibit muscle adhesion and migration in dissociated *Drosophila* embryonic cultures. Ph.D. Thesis.

[56] Li P., Ponnala L., Gandotra N., Wang L., Si Y., Tausta S.L., Kebrom T.H., Provart N., Patel R., Myers C.R. (2010). The developmental dynamics of the maize leaf transcriptome. Nat. Genet..

[57] Eisen M.B., Spellman P.T., Brown P.O., Botstein D. (1998). Cluster analysis and display of genome-wide expression patterns. Proc. Natl. Acad. Sci. USA.

[58] Ramoni M.F., Sebastiani P., Kohane I.S. (2002). Cluster analysis of gene expression dynamics. Proc. Natl. Acad. Sci. USA.

[59] Wang M., Liu C., Su Y., Zhang K., Zhang Y., Chen M., Ge M., Gu L., Lu T., Li N. (2017). MiRNA-34c inhibits myoblasts proliferation by targeting YY1. Cell Cycle.

[60] Zhang R.P., Liu H.H., Wang H.H., Wang Y., Han C.C., Li L., He H., Xu H.Y., Xu F., Wang J.W. (2014). Silencing Pax3 by shRNA inhibits the proliferation and differentiation of duck (*Anas platyrhynchos*) myoblasts. Mol. Cell. Biochem..

[61] Song C., Wu G., Xiang A., Zhang Q., Li W., Yang G., Shi X., Sun S., Li X. (2015). Over-expression of miR-125a-5p inhibits proliferation in C2C12 myoblasts by targeting E2F3. Chin. J. Biochem. Mol. Biol..

[62] Wang H., Jint H., Liu H., Sun L., Li X., Yang C., Zhang R., Li L., Wang J. (2014). Molecular cloning and expression pattern of duck Six1 and its preliminary functional analysis in myoblasts transfected with eukaryotic expression vector. Indian J. Biochem. Biophys..

[63] Panda A.C., Kotb A., Martindale J.L., Clara D.G., Yang X., Ioannis G., Heon N.J., Zhang Y., Elin L., Dudekula D.B. (2016). Novel RNA-binding activity of MYF5 enhances Ccnd1/Cyclin D1 mRNA translation during myogenesis. Nucleic Acids Res..

[64] Rogers M.A., Kalter V., Strowitzki M., Schneider M., Lichter P. (2016). IGF2 knockdown in two colorectal cancer cell lines decreases survival, adhesion and modulates survival-associated genes. Tumour Biol..

[65] Girona J., Rosales R., Plana N., Saavedra P., Masana L., Vallvé J.C. (2013). FABP4 induces vascular smooth muscle cell proliferation and migration through a MAPK-dependent pathway. PLoS ONE.

[66] Wan J., Su Y., Song Q., Tung B., Oyinlade O., Liu S., Ying M., Ming G.L., Song H., Qian J. (2017). Methylatedcis-regulatory elements mediate KLF4-dependent gene transactivation and cell migration. eLIFE.

[67] Clemente C.F.M.Z., Corat M.A.F., Saad S.T.O., Franchini K.G. (2005). Differentiation of C2C12 myoblasts is critically regulated by fak signaling. Am. J. Physiol. Regul. Integr. Comp. Physiol..

[68] Yang F.C., Kapur R., King A.J., Tao W., Kim C., Borneo J., Breese R., Marshall M., Dinauer M.C., Williams D.A. (2000). Rac2 stimulates Akt activation affecting BAD/Bcl-XL expression while mediating survival and actin function in primary mast cells. Immunity.

[69] Millay D.P., Gamage D.G., Quinn M.E., Min Y.L., Mitani Y., Basselduby R., Olson E.N. (2016). Structure-function analysis of myomaker domains required for myoblast fusion. Proc. Natl. Acad. Sci. USA.

